# A multiple kernel learning algorithm for drug-target interaction prediction

**DOI:** 10.1186/s12859-016-0890-3

**Published:** 2016-01-22

**Authors:** André C. A. Nascimento, Ricardo B. C. Prudêncio, Ivan G. Costa

**Affiliations:** Center of Informatics, UFPE, Recife, Brazil; Department of Statistics and Informatics, UFRPE, Recife, Brazil; IZKF Computational Biology Research Group, Institute for Biomedical Engineering, RWTH Aachen University Medical School, Aachen, Germany; Aachen Institute for Advanced Study in Computational Engineering Science (AICES), RWTH Aachen University, Aachen, Germany

**Keywords:** Artificial intelligence, Supervised machine learning, Kernel methods, Multiple kernel learning, Drug discovery

## Abstract

**Background:**

Drug-target networks are receiving a lot of attention in late years, given its relevance for pharmaceutical innovation and drug lead discovery. Different in silico approaches have been proposed for the identification of new drug-target interactions, many of which are based on kernel methods. Despite technical advances in the latest years, these methods are not able to cope with large drug-target interaction spaces and to integrate multiple sources of biological information.

**Results:**

We propose KronRLS-MKL, which models the drug-target interaction problem as a link prediction task on bipartite networks. This method allows the integration of multiple heterogeneous information sources for the identification of new interactions, and can also work with networks of arbitrary size. Moreover, it automatically selects the more relevant kernels by returning weights indicating their importance in the drug-target prediction at hand. Empirical analysis on four data sets using twenty distinct kernels indicates that our method has higher or comparable predictive performance than 18 competing methods in all prediction tasks. Moreover, the predicted weights reflect the predictive quality of each kernel on exhaustive pairwise experiments, which indicates the success of the method to automatically reveal relevant biological sources.

**Conclusions:**

Our analysis show that the proposed data integration strategy is able to improve the quality of the predicted interactions, and can speed up the identification of new drug-target interactions as well as identify relevant information for the task.

**Availability:**

The source code and data sets are available at www.cin.ufpe.br/~acan/kronrlsmkl/.

**Electronic supplementary material:**

The online version of this article (doi:10.1186/s12859-016-0890-3) contains supplementary material, which is available to authorized users.

## Background

Drug-target networks are receiving a lot of attention in late years, given their relevance for pharmaceutical innovation and drug repositioning purposes [1–3]. Although the amount of known interactions between drugs and target proteins has been increasing, the number of targets for approved drugs is still only a small proportion (<10 *%*) from the human proteome [1]. Recent advances on high-throughput methods provide ways for the production of large data sets about molecular entities as drugs and proteins. There is also an increase in the availability of reliable databases integrating information about interactions between these entities. Nevertheless, as the experimental verification of such interactions does not scale with the demand for innovation, the use of computational methods for the large scale prediction is mandatory. There is also a clear need for systems-based approaches to integrate these data for drug discovery and repositioning applications [1].

Recently, an increasing number of methods have been proposed for drug-target interaction (DTI) prediction. They can be categorized in ligand-based, docking-based, or network-based methods [4]. The docking approach, which can provide accurate estimates to DTIs, is computationally demanding and requires a 3D model of the target protein. Ligand-based methods, such as the quantitative structure activity relationship (QSAR), are based on a comparison of a candidate ligand to the known ligands of a biological target [5]. However, the utility of these ligand-based methods is limited when there are few ligands for a given target [2, 4, 6]. Alternatively, network based approaches use computational methods and known DTIs to predict new interactions [4, 5]. Even though ligand-based and docking-based methods are more precise when compared to network based approaches, the latter are more adequate for the estimation of new interactions from complete proteomes and drugs catalogs [1]. Therefore, it can indicate novel candidates to be evaluated by more accurate methods.

**Fig. 1:**
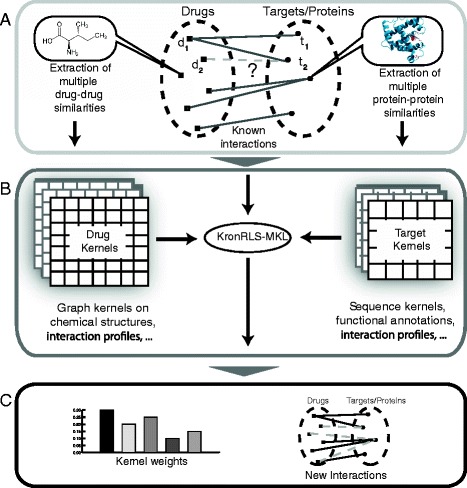
Overview of the proposed method. **a** The drug-target is a bipartite graph with drugs (left) and proteins (right). Edges between drugs and proteins (solid line) indicates a known drug-protein interaction. The drug-protein interaction problem is defined as finding unknown edges (dashed lines) with the assumption that similar drugs (or proteins) should share the same edges. **b** KronRLS-MKL uses several drugs (and protein) kernels to solve the drug-target interaction problem. Distinct Kernels are obtained by measuring similarities of drugs (or proteins) using distinct information sources. **c** KronRLS-MKL provides not only novel predicted interactions as it indicates the relevance (weights) of each kernel used in the predictions

Most network approaches are based on bipartite graphs, in which the nodes are composed of drugs (small molecules) and biological targets (proteins) [3, 7, 8]. Edges between drugs and targets indicate a known DTI (Fig.1). Given a known interaction network, kernel based methods can be used to predict unknown drug-target interactions [2, 9–11]. A *kernel* can be seen as a similarity matrix estimated on all pairs of instances. The main assumption behind network kernel methods is that similar ligands tend to bind to similar targets and vice versa. These approaches use *base kernels* to measure the similarity between drugs (or targets) using distinct sources of information (e.g., structural, pharmacophore, sequence and function similarity). A *pairwise kernel* function, which measures the similarity between drug-target pairs, is obtained by combining a drug and a protein base kernel via kernel product.

The majority of previous network approaches use classification methods, as Support Vector Machines (SVM), to perform predictions over the drug-target interaction space [2, 4]. However, such techniques have major limitations. First, they can only incorporate one pair of base kernels at a time (one for drugs and one for proteins) to perform predictions. Second, the computation of the pairwise kernel matrix for the whole interaction space (all possible drug-target pairs) is computationally unfeasible even for a moderate number of drugs and targets. Moreover, most drug target interaction databases provide no true negative interaction examples. The common solution for these issues is to randomly sample a small proportion of unknown interactions to be used as negative examples. While this approach provides a computationally trackable small drug-target pairwise kernel, it generates an easier but unreal classification task with balanced class size [12].

An emerging machine learning (ML) discipline focused on the search for an optimal combination of kernels, called Multiple Kernel Learning (MKL) [13]. MKL-like methods have been previously proposed to the problem of DTI prediction [14–16] and the closely related protein-protein interaction (PPI) prediction problem [17, 18]. This is extremely relevant, as it allows the use of distinct sources of biological information to define similarities between molecular entities. However, since traditional MKL methods are SVM-based [13, 19], they are subject to memory limitations imposed by the pairwise kernel, and are not able to perform predictions in the complete drugs vs. protein space. Moreover, MKL approaches used in PPI prediction problem [17, 18] and protein function prediction [20, 21] can not be applied to bipartite graphs, as the problem at hand. Currently, we are only aware of two recent works [19, 22] proposing MKL approach to integrate similarity measures for drugs and targets.

Drug-target prediction fits a link prediction problem [4], which can be solved by a Kronecker regularized least squares approach (KronRLS) [10]. A single kernel version of this method has been recently applied to drug-target prediction problem [10, 11]. A recent survey indicated that KronRLS outperforms SVM based methods in DTI prediction [2]. KronRLS uses Kronecker product algebraic properties to be able to perform predictions on the whole drug-target space, without the explicit calculation of the pairwise kernels. Therefore, it can cope with problems on large drugs vs. proteins spaces. However, KronRLS can not be used on a MKL context.

In this work, we propose a new MKL algorithm to automatically select and combine kernels on a bipartite drug-protein prediction problem, the KronRLS-MKL algorithm (Fig 1). For this, we extend the KronRLS method to a MKL scenario. Our method uses *L*2 regularization to produce a non-sparse combination of base kernels. The proposed method can cope with large drug vs. target interaction matrices; does not requires sub-sampling of the drug-target network; and is also able to combine and select relevant kernels. We perform an empirical analysis using drug-target datasets previously described [23] and a diverse set of drug kernels (10) and protein kernels (10).

In our experiments, we considered three different scenarios in the DTI prediction [2, 11, 24]: pair prediction, where every drug and target in the training set have at least one known interaction; or the ‘new drug’ and ‘new target’ setting, where some drugs and targets are present only in the test set, respectively. A comparative analysis with top performance single kernel approaches [2, 8, 10, 25–27] and all competing integrative approaches [14, 15, 22] demonstrates that our method is better or competitive in the majority of evaluated scenarios. Moreover, KronRLS-MKL was able to select and also indicate the relevance of kernels, in the form of weights, for each problem.

## Methods

In this work, we propose an extension of the KronRLS algorithm under recent developments of the MKL framework [28] to address the problem of link prediction on bipartite networks with multiple kernels. Before introducing our method, we will describe the RLS and the KronRLS algorithms (for further information, see [10, 11]).

### RLS and KronRLS

Given a set of drugs $\phantom {\dot {i}\!}D = \{ d_{1}, \ldots, d_{n_{d}}\}$, targets $\phantom {\dot {i}\!}T = \{ t_{1}, \ldots, t_{n_{t}}\}$, and the set of training inputs *x*_*i*_ (drug-target pairs) and their binary labels $y_{i} \in \mathbb {R}$ (where 1 stands for a known interaction and 0 otherwise), with 1<*i*≤*n*, *n*=|*D*||*T*| (number of drug-target pairs). The RLS approach minimizes the following function [29]:

(1) $$ J(f) = \frac{1}{2n}\sum\limits_{i=1}^{n}(y_{i} - f(x_{i}))^{2} + \frac{\lambda}{2} \parallel f {\parallel_{K}^{2}} \;,  $$

where ∥*f*∥_*K*_ is the norm of the prediction function *f* on the Hilbert space associated to the kernel *K*, and *λ*>0 is a regularization parameter which determines the compromise between the prediction error and the complexity of the model. According to the representer theorem [30], a minimizer of the above objective function admits a dual representation of the following form

(2) $$ f(x) = \sum\limits_{i=1}^{n} a_{i} K(x,x_{i}) \;,  $$

where $K: |D||T| \times |D||T| \rightarrow \mathbb {R}$ is named the pairwise kernel function and ***a*** is the vector of dual variables corresponding to each separation constraint. The RLS algorithm obtains the minimizer of Eq. 1 solving a system of linear equations defined by (*K*+*λI*)***a***=***y***, where ***a*** and ***y*** are both *n*-dimensional vectors consisting of the parameters *a*_*i*_ and labels *y*_*i*_.

One can construct such pairwise kernel as the product of two base kernels, namely *K*((*d,t*),(*d*^′^,*t*^′^))=*K*_*D*_(*d,d*^′^)*K*_*T*_(*t,t*^′^), where *K*_*D*_ and *K*_*T*_ are the base kernels for drugs and targets, respectively. This is equivalent to the Kronecker product of the two base kernels [4, 31]: *K*=*K*_*D*_⊗*K*_*T*_. The size of the kernel matrix makes the model training computationally unfeasible even for moderate number of drugs and targets [4].

The KronRLS algorithm is a modification of RLS, and takes advantage of two specific algebraic properties of the Kronecker product to speed up model training: the so called *vec trick* [31] and the relation of the eigendecomposition of the Kronecker product to the eigendecomposition of its factors [11, 32].

Let $K_{D} = Q_{D} \Lambda _{D} {Q_{D}^{T}}$ and $K_{T} = Q_{T} \Lambda _{T} {Q_{T}^{T}}$ be the eigendecomposition of the kernel matrices *K*_*D*_ e *K*_*T*_. The solution ***a*** can be given by solving the following equation [11]:

(3) $$ \boldsymbol{a} = vec(Q_{T} C {Q_{D}^{T}}) \;,  $$

where *vec*(·) is the vectorization operator that stacks the columns of a matrix into a vector, and *C* is a matrix defined as:

(4) $$ C = (\Lambda_{D} \otimes \Lambda_{T} + \lambda I)^{-1}vec({Q_{T}^{T}} Y^{T} Q_{D}) \;.   $$

The KronRLS algorithm is well suited for the large pairwise space involved on the DTI prediction problem, since the estimation of vector ***a*** using Eqs. 3 and 4 is a much faster solution compared to the original RLS estimation process in such scenario. However, it does not support the use of multiple kernels.

### KronRLS MKL

In this work, a vector of different kernels is considered, i.e., $\boldsymbol {k}_{D} = ({K_{D}^{1}}, {K_{D}^{2}},\ldots, K_{D}^{P_{D}})$ and $\boldsymbol {k}_{T} = ({K_{T}^{1}}, {K_{T}^{2}}, \ldots, K_{T}^{P_{T}})$, *P*_*D*_ and *P*_*T*_ indicate the number of base kernels defined over the drugs and target set, respectively. In this section, we propose an extension of KronRLS to handle multiple kernels.

The kernels can be combined by a linear function, i.e., the weighted sum of base kernels, corresponding to the optimal kernels $K_{D}^{*}$ and $K_{T}^{*}$:

$$K_{D}^{*} = \sum\limits_{i=1}^{P_{D}} {\beta_{D}^{i}} {K_{D}^{i}} \; \;, \; \; K_{T}^{*} = \sum\limits_{j=1}^{P_{T}} {\beta_{T}^{j}} {K_{T}^{j}}, $$

where $\boldsymbol {\beta }_{D} = \left \{{\beta _{D}^{1}},\ldots,\beta _{D}^{P_{D}}\right \}$ and $\boldsymbol {\beta }_{T} = \left \{{\beta _{T}^{1}},\ldots,\beta _{T}^{P_{T}}\right \}$, correspond to the weights of drug and protein kernels, respectively.

In [28], the author demonstrated that MKL can be interpreted as a particular instance of a kernel machine with two layers, in which the second layer is a linear function. His work provides the theoretical basis for the development of a MKL extension for the closely related KronRLS algorithm in our work.

The classification function of Eq. 2 can be written in matricial form, *f*_*a*_=*K****a*** [29] and applying the well known property of the Kronecker product, (*A*⊗*B*)*vec*(*X*)=*vec*(*BXA*^*T*^)[32], we have:

$$\begin{array}{*{20}l} f_{a}(X) &= K \boldsymbol{a} \\ &= \left(K_{D}^{*} \otimes K_{T}^{*}\right) vec\left(Q_{T} C {Q_{D}^{T}}\right) \\ &= \left(K_{T}^{*} \left(Q_{T} C {Q_{D}^{T}}\right) \left(K_{D}^{*}\right)^{T}\right). \end{array} $$

This way, we can rewrite the classification function as $\left (K_{T}^{*} A \left (K_{D}^{*}\right)^{T}\right)$, where *A*=*unvec*(***a***). Using the same iterative approach considered in previous MKL strategies [13], we propose the use of a two step optimization process, in which the optimization of the vector ***a*** is interleaved with the optimization of the kernel weights. Given two initial weight vectors, $\boldsymbol {\beta }_{D}^{0}$ and $\boldsymbol {\beta }_{T}^{0}$, an optimal value for the vector ***a***, using Eq. 3 is found, and with such optimal ***a***, we can proceed to find optimal ***β***_*D*_ and ***β***_*T*_. More specifically, Eq. 1 can be redefined when ***a*** is fixed, and knowing that $\parallel f {\parallel _{F}^{2}}=\boldsymbol {a}^{T}K\boldsymbol {a}$ [28], we have:

$$\boldsymbol{u} = \left(\boldsymbol{y} - \frac{\lambda \boldsymbol{a}}{2}\right), $$

then,

(5) $$ J(f_{a}) = \frac{1}{2 \lambda n}\parallel \boldsymbol{u} - K\boldsymbol{a} {\parallel_{2}^{2}} + \frac{1}{2}\boldsymbol{a}^{T} (y-\lambda\boldsymbol{a}).  $$

Since the second term does not depend on *K* (and thus does not depend on the kernel weights), and, as ***y*** and ***a*** are fixed, it can be discarded from the weights optimization procedure. Note that we are not interested in a sparse selection of base kernels as in [28], therefore we introduce a *L*2 regularization term to control sparsity [33] of the kernel weights, also known as a ball constraint. This term is parameterized by the *σ* regularization coefficient. Additionally, we can convert ***u*** to its matrix form by the application of the *unvec* operator, i.e., *U*=*unvec*(***u***), and also use a more appropriate matrix norm (Frobenius, ∥*A*∥_2_≤∥*A*∥_*F*_ [32]). In this way, for any fixed values of ***a*** and ***β***_*T*_, the optimal value for the combination vector is obtained by solving the optimization problem defined as:

(6) $$\begin{array}{*{20}l} & \underset{\boldsymbol{\beta}_{D}}{\text{min}} \;\;\;\frac{1}{2\lambda n}\parallel U - \boldsymbol{m}_{D} \boldsymbol{\beta}_{D} \parallel_{F} + \; \sigma \parallel \boldsymbol{\beta}_{D} {\parallel_{2}^{2}} \end{array} $$

(7) $$\begin{array}{*{20}l} & \boldsymbol{m}_{D} = \left(K_{T}^{*} A \left({K_{D}^{1}}\right)^{T}, K_{T}^{*} A \left({K_{D}^{2}}\right)^{T},\ldots, K_{T}^{*} A\left(K_{D}^{P_{A}}\right)^{T}\right) \end{array} $$

while the optimal ***β***_*T*_ can be found fixing the values of ***a*** and ***β***_*D*_, according to:

(8) $$\begin{array}{*{20}l} & \underset{\boldsymbol{\beta}_{T}}{\text{min}} \;\;\;\frac{1}{2\lambda n}\parallel U - \boldsymbol{\beta}_{T} \boldsymbol{m}_{T} \parallel_{F} + \; \sigma \parallel \boldsymbol{\beta}_{T} {\parallel_{2}^{2}} \end{array} $$

(9) $$\begin{array}{*{20}l} & \boldsymbol{m}_{T} = \left({K_{T}^{1}} A \left(K_{D}^{*}\right)^{T}, {K_{T}^{2}} A \left(K_{D}^{*}\right)^{T},..., K_{T}^{P_{T}} A \left(K_{D}^{*}\right)^{T} \right). \end{array} $$

The optimization method used here is the interior-point optimization algorithm [34] implemented in MATLAB [35].

### Data

The datasets considered were first proposed by [23] and used by most competing methods [2, 10, 11, 15, 25]. Each dataset consists of a binary matrix, containing the known interactions of a determined set of drug targets, namely Enzyme (E), Ion Channel (IC), GPCR and Nuclear Receptors (NR), based on information extracted from the KEGG BRITE [36], BRENDA [37], SuperTarget [38] and DrugBank databases [39]. All four datasets are extremely unbalanced, if we consider the whole drug-target interaction space, i.e., the number of known interactions is extremely lower than the number of unknown interactions, as presented in Table 1.

**Table 1:** Number drugs, targets and positive instances (known interactions) vs. the number of negative (or unknown) interactions on each dataset

	Datasets			
	Nuclear receptors	GPCR	Ion channel	Enzyme
Interactions				
Known	90	635	1476	2926
	(6.41 %)	(3 %)	(3.45 %)	(1 %)
Unknown	1314	20550	41364	292554
	(93.59 %)	(97 %)	(96.55 %)	(99 %)
Entity				
Drugs	54	223	210	445
Targets	26	95	204	664

In order to analyze each type of entity from different points of view, we extracted 20 (10 for targets and 10 for drugs) distinct kernels from chemical structures, side-effects, amino acid sequence, biological function, PPI interactions and network topology (a summary of base kernels is presented in Table 2).

**Table 2:** Network entities and respective kernels considered for combination purposes

Entity	Kernels	Information
		source
Drugs	AERS-bit - AERS bit	Side-effects
	AERS-freq - AERS freq	Side-effects
	GIP - Gaussian Interaction Profile	Network
	LAMBDA - Lambda-k Kernel	Chem. Struct.
	MARG - Marginalized Kernel	Chem. Struct.
	MINMAX - MinMax Kernel	Chem. Struct.
	SIMCOMP - Graph kernel	Chem. Struct.
	SIDER - Side-effects Similarity	Side-effects
	SPEC - Spectrum Kernel	Chem. Struct.
	TAN - Tanimoto Kernel	Chem. Struct.
Proteins	GIP - Gaussian Interaction Profile	Network
	GO - Gene Ontology Semantic Similarity	Func. Annot.
	MIS-k3m1 - Mismatch kernel (*k*=3,*m*=1)	Sequences
	MIS-k4m1 - Mismatch kernel (*k*=4,*m*=1)	Sequences
	MIS-k3m2 - Mismatch kernel (*k*=3,*m*=2)	Sequences
	MIS-k4m2 - Mismatch kernel (*k*=3,*m*=2)	Sequences
	PPI - Proximity in protein-protein network	Protein-protein Interactions
	SPEC-k3 - Spectrum kernel (*k*=3)	Sequences
	SPEC-k4 - Spectrum kernel (*k*=4)	Sequences
	SW - Smith-Waterman aligment score	Sequences

#### Protein kernels

Here we use the following information sources about target proteins: amino acid sequence, functional annotation and proximity in the protein-protein network. Concerning sequence information, we consider the normalized score of the Smith-Waterman alignment of the amino acid sequence (SW) [23], as well as different parametrizations of the Mismatch (MIS) [40] and the Spectrum (SPEC) [41] kernels. For the Mismatch kernel, we evaluated four combinations of distinct values for the k-mers length (*k*=3 and *k*=4) and the number of maximal mismatches per k-mer (*m*=1 and *m*=2), namely MIS-k3m1, MIS-k3m2, MIS-k4m1 and MIS-k4m2; for the Spectrum kernel, we varied the k-mers length (*k*=3 and *k*=4, SPEC-k3 and SPEC-k4, respectively). Both Mismatch and Spectrum kernels were calculated using the R packageKeBABS [42].

The Gene Ontology semantic similarity kernel (GO) was used to encode functional information. GO terms were extracted from the BioMART database [43], and the semantic similarity scores between the GO annotation terms were calculated using the csbl.go R package [44], with the Resnik algorithm [45]. We also extracted a similarity measure from the human protein-protein network (PPI), obtained from the BioGRID database [46]. The similarity between each pair of targets was calculated based on the shortest distance on the corresponding PPI network, according to:

$$S(p,p') = A e^{b D(p,p')}, $$

where *A* and *b* parameters were set as in [14] (*A*=0.9,*b*=1), and *D*(*p,p*^′^) is the shortest hop distance between proteins *p* and *p*^′^.

#### Drug kernels

As drug information sources, we consider 6 distinct chemical structure and 3 side-effects kernels. Chemical structure similarity between drugs was achieved by the application of the SIMCOMP algorithm [47] (obtained from [23]), defined as the ratio of common substructures between two drugs based on the chemical graph alignment. We also computed the Lambda-k kernel (LAMBDA) [48], the Marginalized kernel [49] (MARG), the MINMAX kernel [50], the Spectrum kernel [48] (SPEC) and the Tanimoto kernel [50] (TAN). These later kernels were calculated with the R Package Rchemcpp [48] with default parameters.

Two distinct side-effects data sources were also considered. The FDA adverse event reporting system (AERS), from which side effect keywords (adverse event keywords) similarities for drugs were first retrieved by [51]. The authors introduced two types of pharmacological profiles for drugs, one based on the frequency information of side effect keywords in adverse event reports (AERS-freq) and another based on the binary information (presence or absence) of a particular side-effect in adverse event reports (AERS-bit). Since not every drug in the Nuclear Receptors, Ion Channel, GPCR and Enzyme datasets is also present on AERS-based data, we extracted the similarities of the drugs in AERS, and assigned zero similarity to drugs not present.

The second side-effect resource was the SIDER database^1^ [52]. This database contains information about commercial drugs and their recorded side effects or adverse drug reactions. Each drug is represented by a binary profile, in which the presence or absence of each side effect keyword is coded 1 or 0, respectively. Both AERS and SIDER based profile similarities were obtained by the weighted cosine correlation coefficient between each pair of drug profiles [51].

#### Network topology information

We also use drug-target network structure in the form of a network interaction profile as a similarity measure for both proteins and drugs. The idea is to encode the connectivity behavior of each node in the subjacent network. The Gaussian Interaction Profile kernel (GIP) [10] was calculated for both drugs and targets.

### Competing methods

We compare the predictive performance of the KronRLS-MKL algorithm against other MKL approaches, as well as in a single kernel context (one kernel for drugs, and one for targets). In the latter, we evaluate the performance of each possible combination of base kernels (Table 2) with the KronRLS algorithm, recently reported as the best method for predicting drug-target pairs with single paired kernels [2]. This resulted in a total of 10×10=100 different combinations. The best performing pairs were then used as baselines in our method evaluation, selected according to two distinct criteria: the kernel pair that achieved the largest area under the precision recall curve (AUPR) on the training set, and, a more optimistic approach, which considered the largest AUPR on the testing set.

Besides the combination of single kernels for drugs and targets, two different kinds of methods were adopted to integrate multiple kernels: (1) standard non-MKL kernel methods for DTI prediction, trained on the average of multiple kernels (respectively for drugs and targets); (2) actual MKL methods specifically proposed for DTI prediction.

#### Non-MKL approaches

We extend state-of-the-art methods [8, 10, 25–27] for the DTI prediction problem for a multiple kernel context. For this, initially we average multiple kernels to produce a single kernel (respectively for drugs and targets). Once we have a single average kernel (one for drug and one for target), we adopt a standard kernel method for DTI prediction, i.e., the base learner. In our experiments, two distinct previous combinations strategies are used: the mean of base kernels and the kernel alignment (KA) heuristic, previously proposed by [53]. We will briefly describe the base learners, followed by a short overview of the two combination strategies considered.

The Bipartite Local Model (BLM) [26] is a machine learning based algorithm, where drug-target pairs are predicted by the construction of the so called ‘local models’, i.e., a SVM classifier is trained for each drug in the training set, and the same is done for targets. Then, the maximum scores for drugs and targets are used to predict new drug-target interactions. Since BLM demonstrated superior performance than Kernel Regression Method (KRM) [23] in previous studies [2, 26], we did not consider KRM in our experiments.

The Network-based Random Walk with Restart on the Heterogeneous network (NRWRH) [8] algorithm predicts new interactions between drugs and targets by the simulation of a random walk in the network of known drug-target predictions as well as in the drug-drug and protein-protein similarity networks. LapRLS and NetLapRLS are both proposed in [25]. Both are based on the RLS learning algorithm, and perform similarity normalization by the application of the Laplacian operator. Predictions are done for drugs and targets separately, and the final prediction scores are obtained by averaging the prediction result from drug and target spaces.

As said previously, most previous SVM-based methods found on the literature can be reduced to the Pairwise Kernel Method (PKM) [27], with the distinction being made by the kernels used and the adopted combination strategy. PKM starts with the construction of a pairwise kernel, computed from the drug and target similarities. Given two drug-target pairs, (*d,p*) and (*d*^′^,*p*^′^), and the respective drug and target similarities, *K*_*D*_ and *K*_*P*_, the pairwise kernel is given by *K*((*d,p*),(*d*^′^,*p*^′^)=*K*_*D*_(*d,d*^′^)×*K*_*P*_(*p,p*^′^). Once the pairwise matrix is computed, it is then used to train a SVM classifier.

The PKM [27], KronRLS, BLM, NRWRH, LapRLS and NetLapRLS algorithms cannot cope with multiple kernels. For this reason, we consider two simple methods available for kernel combination: the mean of base kernels and the kernel alignment (KA) heuristic [53]. The mean drug kernel is computed as $K_{D}^{*} = 1 / P_{D} \sum _{i=1}^{P_{D}}{K_{D}^{i}}$, and the same can be done for targets, analogously. KA is a heuristic for the estimation of kernel weights based on the notion of kernel alignment [54]. More specifically, the weight vector, ***β***_*D*_ for instance, can be obtained by:

(10) $$ {\beta_{D}^{i}} = \frac{A\left({K_{D}^{i}},\boldsymbol{yy}^{T}\right)}{\sum\limits_{h=1}^{P_{D}} A\left({K_{D}^{h}},\boldsymbol{yy}^{T}\right)},  $$

where ***yy***^*T*^ stands for the ideal kernel and ***y*** being the label vector. The alignment *A*(*K*,***yy***^*T*^) of a given kernel *K* and the ideal kernel ***yy***^*T*^ is defined as:

(11) $$ A\left(K,\boldsymbol{yy}^{T}\right) = \frac{\left\langle K,\boldsymbol{yy}^{T} \right\rangle_{F}}{n\sqrt{\langle K,K \rangle_{F}}},  $$

where $\left \langle K,\boldsymbol {yy}^{T} \right \rangle _{F} = \sum \limits _{i=1}^{n}\sum \limits _{j=1}^{n} (K)_{\textit {ij}} \left (\boldsymbol {yy}^{T}\right)_{\textit {ij}}$. Once such combinations are performed, the resulting drug and protein kernels are then used as input to the learning algorithm. We refer to the mean and KA heuristics appending the -MEAN and -KA, respectively, to each base learner.

#### Multiple kernel approaches

Similarity-based Inference of drug-TARgets (SITAR) [14] constructs a feature vector with the similarity values, where each feature is based on one drug-drug and one gene-gene similarity measure, resulting in a total of *P*_*D*_×*P*_*T*_ features. Each one is calculated by combining the drug-drug similarities between the query drug and other drugs and the gene-gene similarities between the query gene and other target genes across all true drug-target associations. The method also performs a feature selection procedure and yields the final classification scores using a logistic regression classifier.

Gönen and Kaski [22] proposed the Kernelized Bayesian Matrix Factorization with Twin Multiple Kernel Learning (KBMF2MKL) algorithm, extending a previous work [55] to handle multiple kernels. The KBMF2MKL factorizes the drug-target interaction matrix by projecting the drugs and the targets into a common subspace, where the projected drug and target kernels are multiplied. Normally distributed Kernel weights for each subspace projected kernel are then estimated without any constraints. The product of the final combined matrices is then used to make predictions.

Wang et al. [15] proposes to use a simple heuristic to previously combine the drug and target similarities, and then use a SVM classifier to perform the predictions. Only the maximum similarity values of drug and target kernel matrices are selected, resulting in two distinct kernels. They are then used to construct a pairwise kernel, computed from the drug and target similarities. Once the pairwise matrix is computed, it is then used to train a SVM classifier. This procedure is also known as the Pairwise Kernel Method (PKM) [27]. For this reason, we refer to the approach proposed by [15] by PKM-MAX.

The authors in [15] suggest as further work a weighted sum approach. They suggest to learn the optimal convex combination of data sources maximizing the correlation of the obtained kernel matrix with the topology of drug-protein network. This objective can be achieved by solving a linear programming problem, as follows:

$$\underset{\boldsymbol{\beta}_{D}}{\text{max}} \;\;\; \left|corr(K_{D}^{*}, dist)\right|, $$

where $K_{D}^{*}$ correspond to the optimal combination of drug kernel matrices with weight vector ***β***_*D*_, *dist* is the drug-drug distance matrix in the DTI network, and *corr* represents the correlation coefficient. Analogously, the same can be done for targets. We call this method WANG-MKL.

### Experimental setup

Previous work [2, 11, 24] suggest that, in the context of paired input problems, one should consider separately the experiments where the training and test sets share common drugs or proteins. In order to achieve a clear notion of the performance of each method, all competing approaches were evaluated under 5 runs of three distinct 5-fold cross-validation (CV) procedures:

1. ‘new drug’ scenario: it simulates the task of predicting targets for new drugs. In this scenario, the drugs in a dataset were divided in 5 disjoint subsets (folds). Then the pairs associated to 4 folds of drugs were used to train the classifier and the remaining pairs are used to test;
2. ‘new target’ scenario: it corresponds in turn to predicting interacting drugs for new targets. This is analogous to the above scenario, however considering 5 folds of targets;
3. pair prediction: is consists of predicting unknown interactions between known drugs and targets. All drug-target interactionswere split in five folds, from which 4 were used for training and 1 for testing. Some of the competing methods (PKM-based, WANG-MKL and SITAR) were trained with sub-sampled datasets, i.e., we randomly selected the same number of known interactions among the unknown interaction set, since these methods cannot be executed in large networks [2, 4, 14, 15]. Although balanced classes are unlikely in real scenarios, we also performed experiments in context (3), using a sub-sampled test set, obtained by sampling as many negative examples as positive examples [14, 15] from the test fold. This experiment is relevant for comparison to previous work, since most previous studies on drug-target prediction performed under-sampling to evaluate predictive performance (see Additional file 1: Table S1).^2^

The hyperparameters of each competing methods were optimized under a nested CV procedure, using the following values: for the SVM-based methods (PKM, BLM and WANG-MKL), the SVM cost parameter was evaluated under the interval {2^−1^,…,2^3^}; for the KronRLS-based methods, the *λ* parameter was evaluated in the interval {2^−15^,2^−10^,…,2^30^}. The *σ* regularization coefficient of the KRONRLS-MKL algorithm was also optimized in the interval {0,0.25,0.5,0.75,1}. The number of components in KBMF2MKL was varied in the interval *R*∈{5,10,…,40}, and for the LapRLS and NetLapRLS we varied *β*_*d*_,*β*_*t*_∈{0.25,0.50,…,1}. In NetLapRLS we also considered two distinct values for *γ*_*d*2_,*γ*_*t*2_∈{0.01,0.1}. For NRWRH the restart probability was evaluated in the set {0.1,0.2,…,0.9}. After the hyperparameters were selected for each method, the outer loop evaluated the predictive performance for the test set partition with the model built using the selected hyperparameters.

The evaluation metric considered was the AUPR, as it allows a good quantitative estimate of the ability to separate the positive interactions from the negative ones. According to [56], this metric provides a better quality estimate for highly unbalanced data, since it punishes more heavily the existence of false positives (FP). This is specially true for the datasets considered, as demonstrated on Table 1, in which all datasets are extremely unbalanced.

## Results and discussion

### Paired kernel experiments

As a base study, we evaluate the performance of KronRLS on all pairs of kernels (10×10 pairs). The AUPR results of all pairs of kernels for the Nuclear Receptors, GPCR, Ion Channel and Enzyme datasets are show in more detail in the supplementary material (see Additional file 1).

**Fig. 2:**
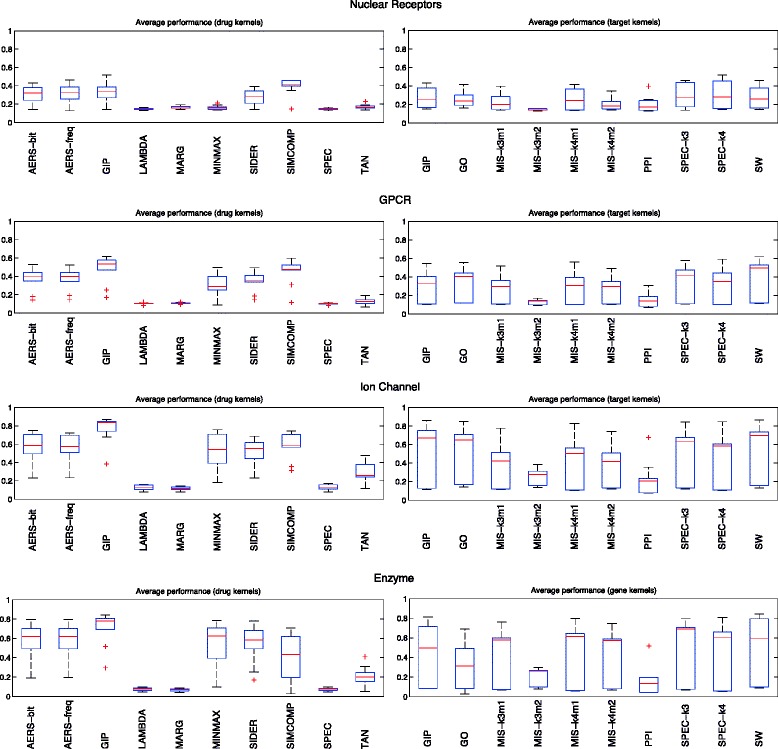
Average performance of each single kernel with the KronRLS algorithm as base learner. The boxplots shows the AUPR performance of drug and protein kernels across different kernel combinations

The performance of KronRLS varies drastically with the kernel choice, as clearly demonstrated by the average performance of each kernel on the single kernel experiments (Fig. 2). For Nuclear Receptors, the best kernel pair combination was SPEC-k4 and GIP, while GIP and SW performed best in all other data sets. It is also important to notice the impact of different parametrizations of the Mismatch sequence kernel. Its performance decreases as more mismatches are allowed inside a k-mer. Overall, both versions of AERS, SIMCOMP, GIP, MINIMAX and SIDER drug kernels showed better performance, while LAMBDA, MARG, SPEC and TAN performed worse. For targets, GIP, GO, MIS-k4m1, SPEC and SW kernels performed better than other target kernels.

### Comparative analysis

In this section, we compare the competing methods in terms of AUPR for all datasets. Concerning KronRLS, we will use the best kernel pair (Best Pair) with largest AUPR as described in the previous section. This will serve as a baseline to evaluate the MKL approaches. Results are presented in Table 3. In the pair prediction scenario, KRONRLS-MKL obtained highest AUPR in all datasets. Its results are even superior than the performance in comparison to the best kernel pair under the optimistic selection. The results of KRONRLS-MKL in pair prediction are statistically significant against all other methods (at *α*=0.05), except from KRONRLS-KA and KRONRLS-MEAN, according to the Wilcoxon rank sum test (Additional file 2). Concerning the subsampled pair prediction, KRONRLS-MKL achieved highest AUPR in the NR and IC data sets, and SITAR performed best in the GPCR and Enzyme data. There it performed second, just after SITAR (see Additional file 3: Table S1). The highest AUPR values obtained in the subsampled data sets in comparison to the unbalanced data sets clearly indicate that performing predictions in the complete data is a more difficult task. Moreover, the number of positive examples was negatively correlated to the dataset size for the complete datasets.

**Table 3:** Results on MKL Experiments on 5 × 5 cross-validation experiments

Dataset	Combination	Pairs		Targets		Drugs	
NR	[SPEC-k4]-[AERS-freq] ^*†*^	0.4630	(±0.0215)	0.3851	(±0.0254)	0.2341	(±0.0054)
	[SPEC-k4]-[GIP] ^∗^	0.5187	(±0.0255)	0.3725	(±0.0247)	0.0949	(±0.0068)
	BLM-KA	0.0709	(±0.0048)	0.3441	(±0.0264)	0.3130	(±0.0224)
	BLM-MEAN	0.0685	(±0.0062)	0.3453	(±0.0264)	0.2934	(±0.0154)
	KBMF2MKL	0.2041	(±0.0150)	0.2059	(±0.0388)	0.1459	(±0.0272)
	KRONRLS-KA	0.4321	(±0.0147)	0.3489	(±0.0337)	0.2850	(±0.0126)
	KRONRLS-MEAN	0.4078	(±0.0211)	0.3482	(±0.0341)	0.2665	(±0.0109)
	KRONRLS-MKL	**0.5368**	(±0.0137)	**0.3541**	(±0.0321)	**0.3383**	(±0.0224)
	LAPRLS-KA	0.1989	(±0.0207)	0.2120	(±0.0277)	0.1841	(±0.0044)
	LAPRLS-MEAN	0.1870	(±0.0196)	0.2008	(±0.0251)	0.1832	(±0.0022)
	NETLAPRLS-KA	0.2310	(±0.0277)	0.2091	(±0.0288)	0.1841	(±0.0044)
	NETLAPRLS-MEAN	0.2195	(±0.0273)	0.1989	(±0.0263)	0.1831	(±0.0023)
	NRWRH-KA	–	–	0.1776	(±0.0380)	0.1911	(±0.0116)
	NRWRH-MEAN	–	–	0.1755	(±0.0364)	0.1881	(±0.0109)
	PKM-KA	0.1830	(±0.0114)	0.2363	(±0.0387)	0.1741	(±0.0158)
	PKM-MAX	0.0946	(±0.0188)	0.0774	(±0.0108)	0.1174	(±0.0080)
	PKM-MEAN	0.1702	(±0.0099)	0.2163	(±0.0400)	0.1672	(±0.0152)
	SITAR	0.4477	(±0.0658)	0.1396	(±0.0505)	0.0694	(±0.0189)
	WANG-MKL	0.3293	(±0.0175)	0.2238	(±0.0300)	0.2628	(±0.0225)
GPCR	[SPEC-k4]-[MINMAX] ^*†*^	0.3246	(±0.0093)	0.5053	(±0.0322)	0.0924	(±0.0055)
	[SW]-[GIP] ^∗^	0.6188	(±0.0075)	0.4561	(±0.0201)	0.0419	(±0.0014)
	BLM-KA	0.0633	(±0.0071)	**0.5508**	(±0.0123)	0.3000	(±0.0198)
	BLM-MEAN	0.0519	(±0.0032)	0.5353	(±0.0135)	0.2526	(±0.0188)
	KBMF2MKL	0.4960	(±0.0124)	0.0963	(±0.0346)	0.1408	(±0.0120)
	KRONRLS-KA	0.6208	(±0.0081)	0.4727	(±0.0101)	0.3005	(±0.0148)
	KRONRLS-MEAN	0.6213	(±0.0085)	0.4461	(±0.0086)	0.2731	(±0.0155)
	KRONRLS-MKL	**0.6440**	(±0.0052)	0.4127	(±0.0126)	**0.3161**	(±0.0112)
	LAPRLS-KA	0.2183	(±0.0067)	0.1458	(±0.0050)	0.1210	(±0.0058)
	LAPRLS-MEAN	0.2169	(±0.0066)	0.1369	(±0.0049)	0.1215	(±0.0061)
	NETLAPRLS-KA	0.3763	(±0.0096)	0.1451	(±0.0041)	0.1211	(±0.0062)
	NETLAPRLS-MEAN	0.3841	(±0.0088)	0.1357	(±0.0039)	0.1221	(±0.0061)
	NRWRH-KA	–	–	0.0762	(±0.0041)	0.1201	(±0.0088)
	NRWRH-MEAN	–	–	0.0704	(±0.0036)	0.1176	(±0.0099)
	PKM-KA	0.2625	(±0.0133)	0.2327	(±0.0175)	0.1424	(±0.0146)
	PKM-MAX	0.1230	(±0.0106)	0.0652	(±0.0071)	0.0935	(±0.0044)
	PKM-MEAN	0.2613	(±0.0178)	0.1632	(±0.0186)	0.1254	(±0.0107)
	SITAR	0.5324	(±0.0267)	0.1151	(±0.0538)	0.0283	(±0.0110)
	WANG-MKL	0.4240	(±0.0071)	0.3521	(±0.0111)	0.2686	(±0.0274)
IC	[PPI]-[GIP] ^*†*^	0.6789	(±0.0078)	0.1548	(±0.0020)	0.0467	(±0.0009)
	[SW]-[GIP] ^∗^	0.8679	(±0.0056)	0.7301	(±0.0140)	0.0476	(±0.0008)
	BLM-KA	0.1169	(±0.0127)	**0.7944**	(±0.0047)	**0.2516**	(±0.0304)
	BLM-MEAN	0.1106	(±0.0088)	0.7798	(±0.0040)	0.2152	(±0.0257)
	KBMF2MKL	0.7671	(±0.0033)	0.4420	(±0.0141)	0.0856	(±0.0044)
	KRONRLS-KA	0.8553	(±0.0017)	0.7246	(±0.0071)	0.2039	(±0.0190)
	KRONRLS-MEAN	0.8693	(±0.0011)	0.6885	(±0.0067)	0.1887	(±0.0186)
	KRONRLS-MKL	0.8769	(±0.0011)	0.6894	(±0.0056)	0.2406	(±0.0259)
	LAPRLS-KA	0.3088	(±0.0021)	0.2747	(±0.0031)	0.0942	(±0.0022)
	LAPRLS-MEAN	0.3187	(±0.0024)	0.2760	(±0.0032)	0.0939	(±0.0021)
	NETLAPRLS-KA	0.5359	(±0.0065)	0.2750	(±0.0032)	0.0931	(±0.0022)
	NETLAPRLS-MEAN	0.5560	(±0.0073)	0.2766	(±0.0034)	0.0928	(±0.0023)
	NRWRH-KA	–	–	0.2371	(±0.0046)	0.0720	(±0.0026)
	NRWRH-MEAN	–	–	0.2363	(±0.0042)	0.0712	(±0.0024)
	PKM-KA	0.5133	(±0.0235)	0.4151	(±0.0092)	0.1156	(±0.0041)
	PKM-MAX	0.1608	(±0.0132)	0.1673	(±0.0038)	0.0660	(±0.0031)
	PKM-MEAN	0.5474	(±0.0261)	0.3840	(±0.0062)	0.0998	(±0.0019)
	SITAR	0.7505	(±0.0153)	0.1717	(±0.0633)	0.0174	(±0.0046)
	WANG-MKL	0.7116	(±0.0214)	0.6009	(±0.0158)	0.2217	(±0.0124)
E	[GO]-[GIP] ^*†*^	0.6900	(±0.0032)	0.2371	(± 0.0025)	0.0124	(±0.0004)
	[SW]-[GIP] ^∗^	0.8429	(±0.00540)	0.7438	(± 0.0189)	0.0159	(±0.0003)
	BLM-KA	0.0471	(±0.0045)	**0.8201**	(±0.0070)	**0.2506**	(±0.0060)
	BLM-MEAN	0.0374	(±0.0032)	0.8099	(±0.0063)	0.2079	(±0.0051)
	KBMF2MKL	0.6722	(±0.0051)	0.0757	(±0.0049)	0.0213	(±0.0004)
	KRONRLS-KA	0.8630	(±0.0127)	0.7274	(±0.0071)	0.1829	(±0.0034)
	KRONRLS-MEAN	0.8667	(±0.0098)	0.6917	(±0.0062)	0.1655	(±0.0030)
	KRONRLS-MKL	0.8818	(±0.0128)	0.7384	(±0.0063)	0.2168	(±0.0050)
	LAPRLS-KA	0.1920	(±0.0014)	0.1677	(±0.0072)	0.0682	(±0.0012)
	LAPRLS-MEAN	0.1750	(±0.0015)	0.1402	(±0.0055)	0.0646	(±0.0013)
	NETLAPRLS-KA	0.2853	(±0.0024)	0.1669	(±0.0042)	0.0670	(±0.0018)
	NETLAPRLS-MEAN	0.2548	(±0.0019)	0.1402	(±0.0046)	0.0636	(±0.0016)
	NRWRH-KA	–	–	0.0886	(±0.0011)	0.0403	(±0.0024)
	NRWRH-MEAN	–	–	0.0816	(±0.0006)	0.0383	(±0.0018)
	PKM-KA	0.2383	(±0.0069)	0.1905	(±0.0047)	0.0480	(±0.0037)
	PKM-MAX	0.0762	(±0.0011)	0.0597	(±0.0007)	0.0323	(±0.0007)
	PKM-MEAN	0.2161	(±0.0072)	0.1239	(±0.0032)	0.0382	(±0.0031)
	SITAR	0.7558	(±0.0160)	0.0232	(±0.0151)	0.0097	(±0.0111)
	WANG-MKL	0.7286	(±0.0046)	0.6663	(±0.0069)	0.1648	(±0.0042)

Best performing methods are indicated in bold. Standart deviation is indicated in brackets. Training of the PKM, SITAR and WANG algorithms was done with the balanced training set

^*†*^best on training

^∗^best on testing

In the ’new target’ scenario, BLM-KA performed best in 3 of 4 datasets, followed closely by BLM-mean and KRONRLS-MKL, demonstrating that the local SVM model is more effective in such scenario. BLM-KA performed better than all evaluated methods with the exception of BLM-Mean, KBMF-MKL, KRONRLS-KA, KRONRL-MEAN and KRONRLS-MKL (*α*=0.05 Additional file 2). In the ’new drug’ problem, KRONRLS-MKL obtained higher AUPR in the NR and GPCR datasets, while BLM-KA had higher AUPR values in the IC and Enzyme data. Both KRONRLS-MKL and BLM-KA had statistically significant higher AUPR (at *α*=0.05; Additional file 2) than all other competing methods. In order to give an overview of the performance of the evaluated methods, an average ranking of the AUPR values obtained by all methods across the four datasets is presented in Table 4.

**Table 4:** Average ranking over all four datasets

	Prediction task		
Method	Pair	Targets	Drugs
SINGLE ^*†*^	7.0	7.8	15.0
SINGLE ^∗^	3.3	3.3	17.5
BLM-KA	16.0	2.5	1.8
BLM-MEAN	17.0	3.0	4.0
KBMF2MKL	7.3	13.5	13.3
KRONRLS-KA	3.8	4.3	3.8
KRONRLS-MEAN	3.0	5.8	5.0
KRONRLS-MKL	1.0	4.8	1.5
LAPRLS-KA	12.8	11.5	9.8
LAPRLS-MEAN	13.3	12.8	10.5
NETLAPRLS-KA	9.3	12.0	10.3
NETLAPRLS-MEAN	9.0	13.0	11.3
NRWRH-KA	–	15.8	12.0
NRWRH-MEAN	–	16.8	13.0
PKM-KA	11.8	8.8	9.8
PKM-MAX	15.0	18.5	16.0
PKM-MEAN	12.0	11.0	11.5
SITAR	5.0	17.3	19.0
WANG-MKL	6.8	7.8	5.0

^*†*^best on training

^∗^best on testing

Methods also displayed distinct computational requirements. Memory usage was stable accross all methods, except from the SVM-based algorithms, which demonstrated quadratic growth of the memory used in relation to the size of the dataset (BLM, PKM, WANG-MKL). This is in part due to the construction of the explicit pairwise kernel (see Additional file 3: Table S3). This fact turns such methods inadequate for contexts in which subsampling of pairs is undesirable.

We now discuss about computational time in the pair prediction scenario. The precomputed kernels approaches (MEAN and KA) were overall the fastest on average, with PKM-based methods requiring less time to train and test the models (∼1 min), followed by KronRLS-based and LapRLS-based algorithms(∼20 and 27 min, respectively). KBMF2MKL and BLM were the slowest, requiring more than 100 min on average at the same task. The lower computation time of the heuristic-based methods is explained by the absence of complex optimization procedures to find the kernel weights. KronRLS-MKL took a little less time than KBMF2MKL, taking an average over the four datasets of 74 min. (see Additional file 3: Table S4).

### Predictions on new drug-target interactions

In order to evaluate the quality of final predictions in a more realistic scenario, we performed an experiment similar to that described by [10, 26]. We estimate the most highly ranked drug–target pairs as most likely true interactions, and performed a search on the current release of four major databases (DrugBank [39], MATADOR [38], KEGG [57]) and ChEMBL [58]. As the training datasets were generated almost eight years ago, new interactions included in these databases will serve as a external validation set. We exclude interactions already present in the training data.

**Fig. 3:**
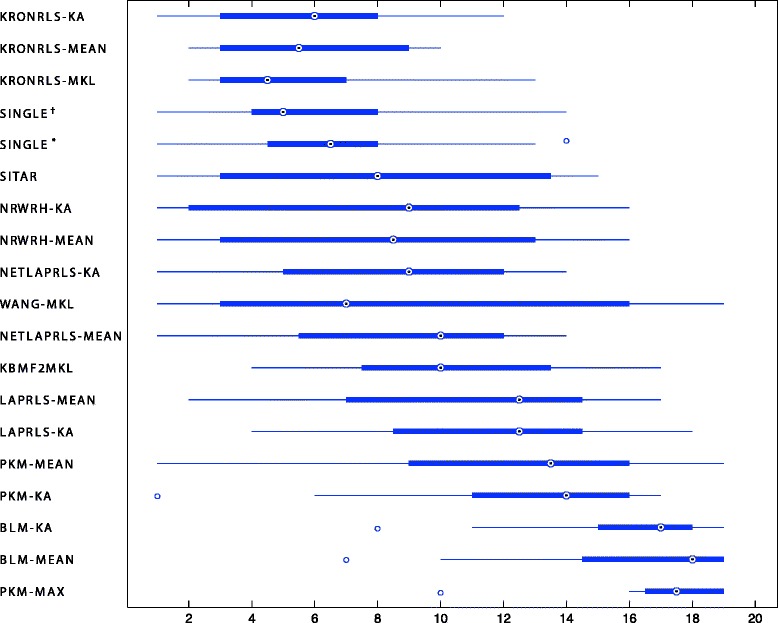
Mean AUPR ranking of each method when compared to the new interactions found on updated databases. The KronRLS-based methods achieved superior performance when compared to other integration strategies

We trained all methods with all interactions present in the original datasets. In the specific case of BLM and NRWRH, one model for drugs and another for targets was trained, and then the maximum score for each DT pair was considered for prediction. Then, we calculated the AUPR for each dataset separately, discarding already known interactions (see Additional file 3: Table S2). The low AUPR values of all methods indicate the difficulty in performing predictions in such large search space. An average ranking (Fig. 3) of each method across all databases indicates that KronRLS methods as best performing algorithms followed by single kernel approaches. It is also important to highlight the poor performance of BLM-KA and BLM-MEAN in this task. This indicates a poor generalization capacity of the BLM framework to the drug-target prediction problem (see Table 3).

Next, a more practical assessment of the predicting power of KRONRLS-MKL is done, by looking to the top 5 ranked interactions predicted by our method (Table 5). We observe that the great majority of interactions (14 out of 20) have been already described in ChEMBL, DrugBank or Matador. We focus our discussion in selected novel interactions. For example, in the Nuclear Receptor database, the 5th ranked prediction indicates the association of Tretinoin with the nuclear factor RAR-related orphan receptor A (RORa). Tretinoin is a drug currently used to treatment of acnes [59]. Interestingly, its molecular activity is associated with the activation of nuclear receptors of the closelly related RAR family.

**Table 5:** Top five predicted interactions by KRONRLS-MKL

Drug		Target		
Nuclear Receptors				
D00951	*Medroxyprogesterone acetate*	hsa2099	*estrogen receptor 1*	(D,C)
D00585	*Mifepristone*	hsa2099	*estrogen receptor 1*	(C)
D00182	*Norethisterone*	hsa2099	*estrogen receptor 1*	(C)
D00105	*Estradiol*	hsa5241	*progesterone receptor*	(C)
D00094	*Tretinoin*	hsa6095	*RAR-related orphan receptor A*	
GPCR				
D02358	*Metoprolol*	hsa154	*adrenoceptor beta 2, surface*	(D,C)
D00283	*Clozapine*	hsa1814	*dopamine receptor D3*	(D,C,M)
D00371	*Theophylline*	hsa135	*adenosine A2a receptor*	(K,D,C)
D00371	*Theophylline*	hsa134	*adenosine A1 receptor*	(K,D,C)
D00095	*Adrenaline*	hsa155	*adrenoceptor beta 3*	(K,D,C)
Ion Channel				
D00775	*Riluzole*	hsa2898	*glutamate receptor, ionotropic, kainate 2*	(M)
D02356	*Verapamil*	hsa6833	*ATP-binding cassette, sub-family C (CFTR*	
D00294	*Diazoxide*	hsa10060	*ATP-binding cassette, sub-family C (CFTR*	
D02356	*Verapamil*	hsa56660	*potassium channel, two pore domain subfamily K, member 12*	
D00524	*Carbachol*	hsa1134	*cholinergic receptor, nicotinic, alpha 1 (muscle)*	
Enzyme				
D00542	*Halothane*	hsa1571	*cytochrome P450, family 2, subfamily E, polypeptide 1*	(D,C,M)
D00437	*Nifedipine*	hsa1559	*cytochrome P450, family 2, subfamily C, polypeptide 9*	(D,C,M)
D00528	*Anhydrous caffeine*	hsa1549	*cytochrome P450, family 2, subfamily A, polypeptide 7*	(M)
D03670	*Deferoxamine*	hsa1579	*cytochrome P450, family 4, subfamily A, polypeptide 11*	
D00139	*Methoxsalen*	hsa1543	*cytochrome P450, family 1, subfamily A, polypeptide 1*	(D,M)

Interactions found in KEGG, DrugBank, ChEMBL and Matador are marked as K, D, C and M respectively

This is also a good example to illustrate the benefits for incorporation of multiple sources of data. Both RORa and Tretinoin do not share nodes in the training set. All targets of Tretinoin have a high GO similarity to RORa (mean value of 0.8368) despite of theirr low sequence similarity (SW mean value is 0.1563). In addition, one of the targets RORa is NR0B1 (nuclear receptor subfamily 0, group B, member 1). This protein is very close to RORa in the PPI network (similarity score of 0.90).

Concerning Ion Channel models, prediction ranked 2 and 3 indicate the interaction of Verapamil and Diazoxide with ATP-binding cassete sub-family C (ABBCC8). ABBCC8 is one of the proteins encoding the sulfonylurea receptor (SUR1) and is associated to calcium regulation and diabetes type I [60]. Interestingly, there are positive reports of Diazoxide treatments to prevent diabetes in rats [61].

### Evaluation of kernel weigths

**Fig. 4:**
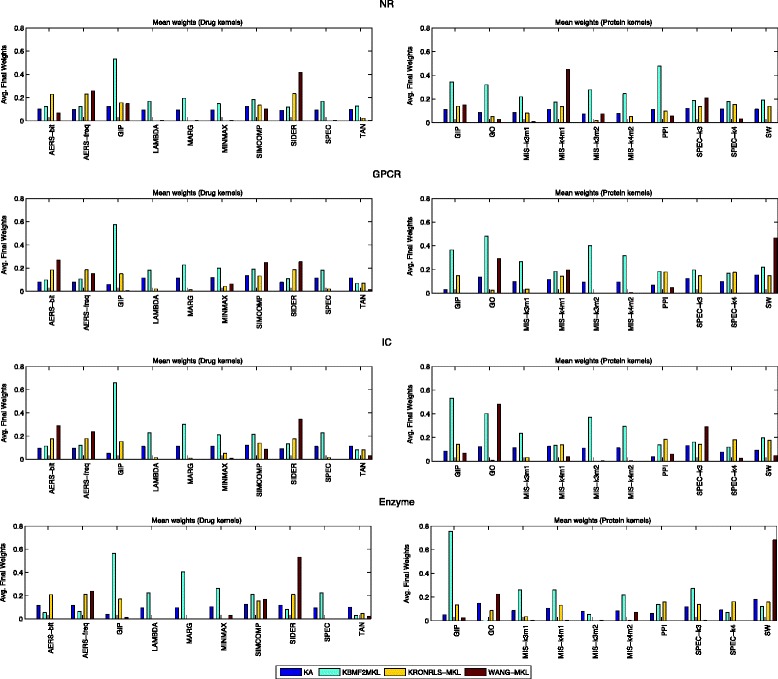
Comparison of the average final weights obtained by the Kernel Alignment (KA) heuristic, KBMF2MKL, KronRLS-MKL and WANG-MKL algorithms. As one can note, the KA heuristic demonstrated close to mean weights, while KRONRLS-MKL and WANG-MKL effectively discarded the most irrelevant kernels

The kernel weights given by KBMF2MKL, KRONRLS-MKL and WANG-MKL, as well as the KA heuristic, can be used to analyze the ability of such methods to identify the most relevant information sources. As there is no guideline or gold standard for this, we resort to a simple approach: compare the kernel weights (Fig. 4) with the average performance of each kernel on the single kernel experiments (Fig. 2). First, it is noticeable that the KA weights are very similar to the average selection (0.10). This indicates that no clear kernel selection is performed. WANG-MKL and KRONRLS-MKL give low weights to drug kernels LAMBDA, MARG, MINIMAX, SPEC and TAN and protein kernel MIS-k3m2. These kernels have overall worst AUPR in the single kernel experiments, which indicates an agreement with both selection procedures. Although the weights assigned by KBMF2MKL are not subject to convex constraints, as indicated by the larger weights assigned to all kernels, they also provide a notion of quality of base kernels. We can observe a stronger preference to the GIP kernel, in all datasets, even though the algorithm assigned a high weight for the lower quality MIS-k3m2 in three of the four datasets.

## Conclusions

We have presented a new Multiple Kernel Learning algorithm for the bipartite link prediction problem, which is able to identify and select the most relevant information sources for DTI prediction. Most previous MKL methods mainly solve the problem of MKL when kernels are built over the same set of entities, which is not the case for the bipartite link prediction problem, e.g. drug-target networks. Regarding predictions in drug-target networks, the sampling of negative/unknown examples, as a way to cope with large data sets, is a clear limitation [2]. Our method takes advantage of the KronRLS framework to efficiently perform link prediction on data with arbitrary size.

In our experiments, the KronRLS-MKL algorithm demonstrated an interesting balance between accuracy and computational cost in relation to other approaches. It performed best in the “pair” prediciton problem and the “new target” problem. In the ’new drug’ and ’new target’ prediction tasks, BLM-KA was also top ranked. This method has a high computational cost. This arises from the fact it requires a classifier for each DT pair [2]. Moreover, it obtained poor results in the evaluation scenario to predict novel drug-protein pairs interactions.

The convex constraint estimation of kernel weights correlated well with the accuracy of a brute force pair kernel search. This non-sparse combination of kernels possibly increased the generalization of the model by reducing the bias for a specific type of kernel. This usually leads to better performance, since the model can benefit from different heterogeneous information sources in a systematic way [33]. Finally, the algorithm performance was not sensitive to class unbalance and can be trained over the whole interaction space without sacrificing performance.

## Endnotes

^1^http://sideeffects.embl.de/.

^2^ NRWRH cannot be applied to the pair prediction [8], by which this method was not considered in such context.

## Additional files

Additional file 1
**Figure.** Single kernel experiments on the Nuclear Receptor dataset with the KronRLS algorithm as base learner. The heatmap shows the AUPR performance of different kernel combinations; red means higher AUPR. (PDF 460 kb)

Additional file 2
**Spreadsheet.** *p*-values under pairwise Wilcoxon Rank Sum statistical tests of all competing methods in pair, drug and target prediction tasks. (XLS 24 kb)

Additional file 3
**Supplementary Tables.** AUPR Results of competing methods under pair prediction setting considering subsampled test sets (S1); AUPR results of predicted scores against new interactions found on current release of KEGG, Matador, Drugbank and ChEMBL databases (S2); Average memory (MB) usage during training and testing of competing methods (S3); Average time (minutes) required to train and test the models with the competing methods (S4). (PDF 89.7 kb)
