# Supplementary figures and images for: A multiple kernel learning algorithm for drug-target interaction prediction
Source: BMC Bioinformatics. 2016 Jan 22;17:46. doi: 10.1186/s12859-016-0890-3 (PMC4722636; doi:10.1186/s12859-016-0890-3)

Nuclear Receptors

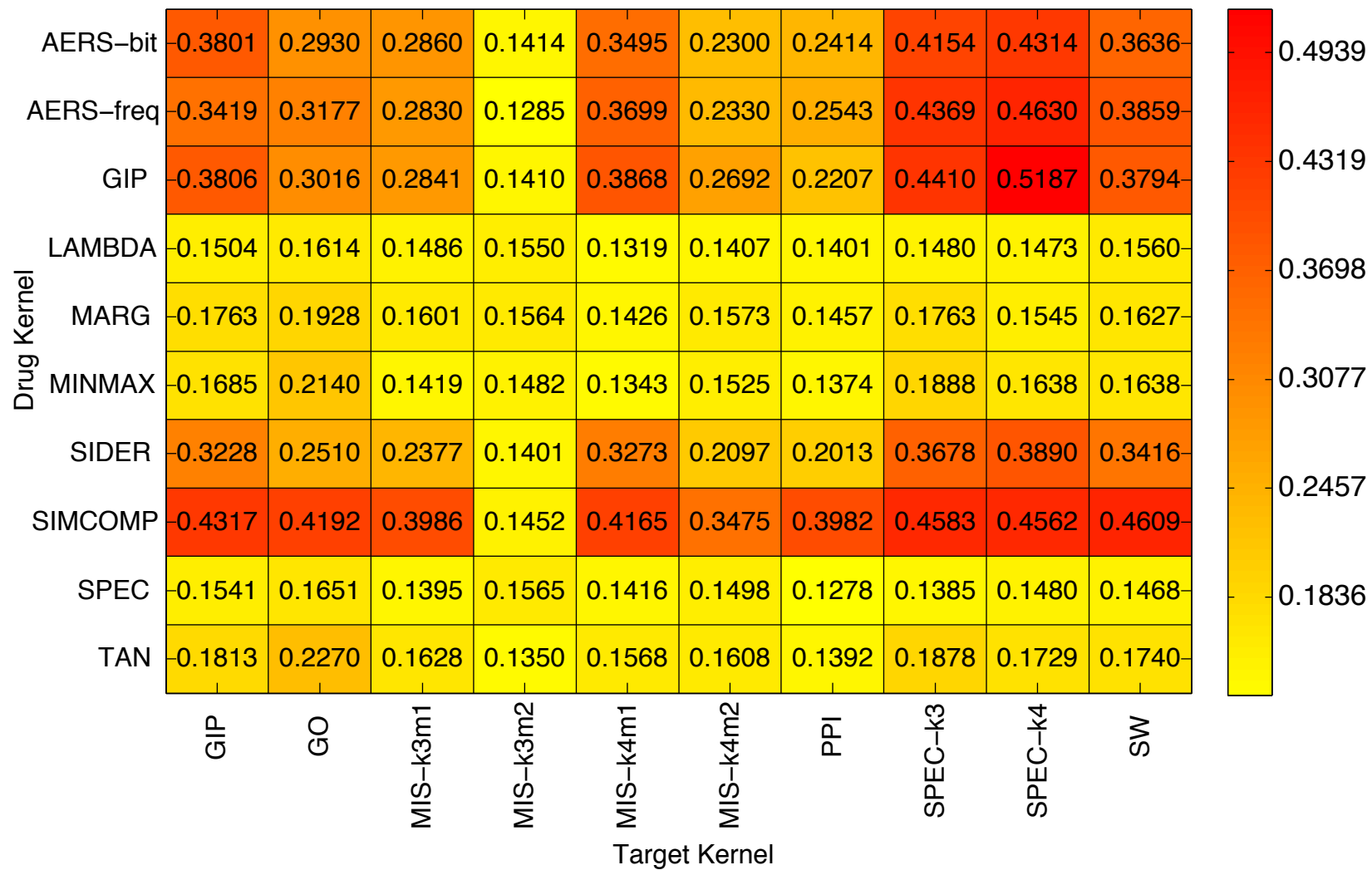

GPCR

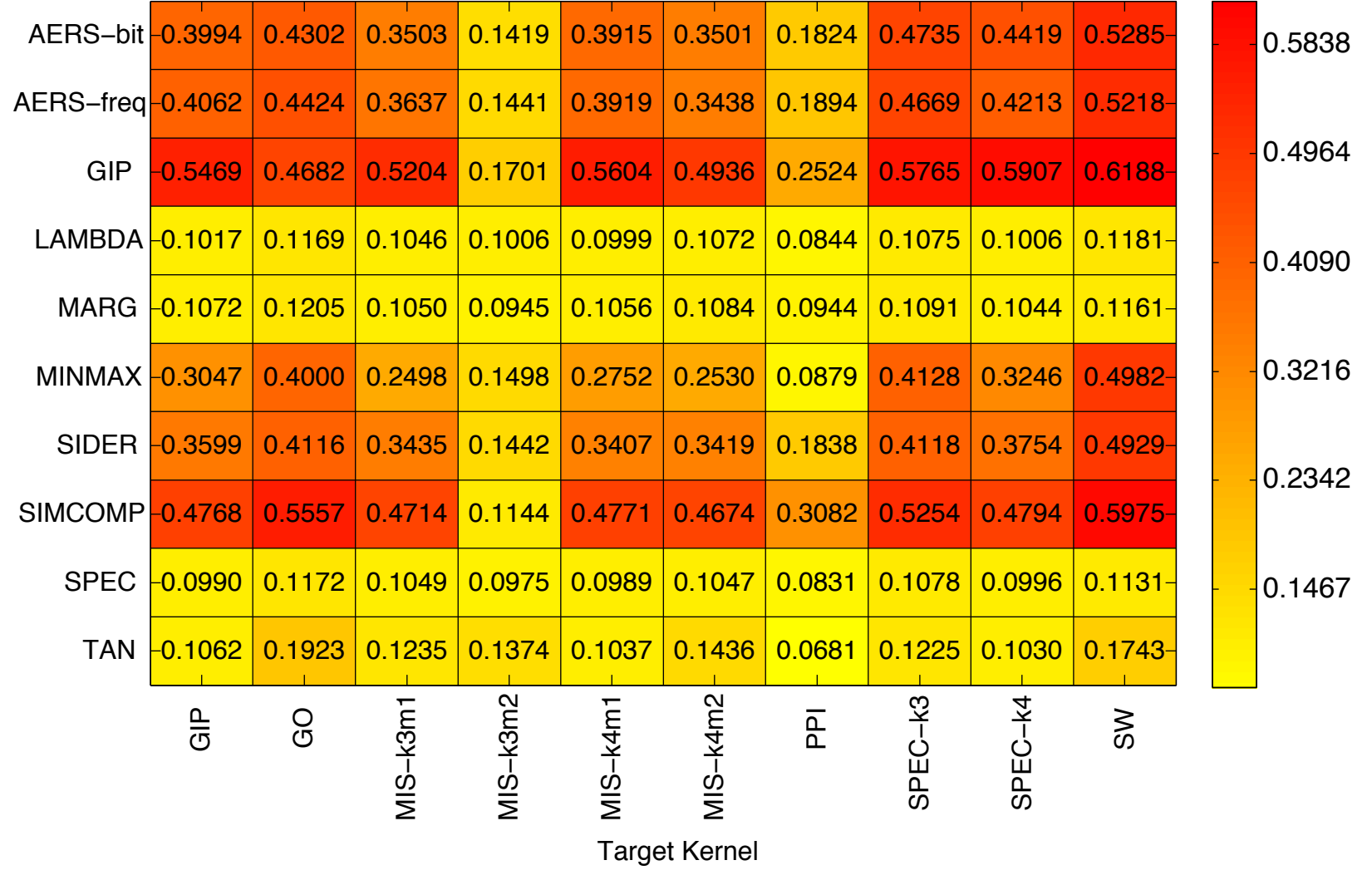

Ion Channel

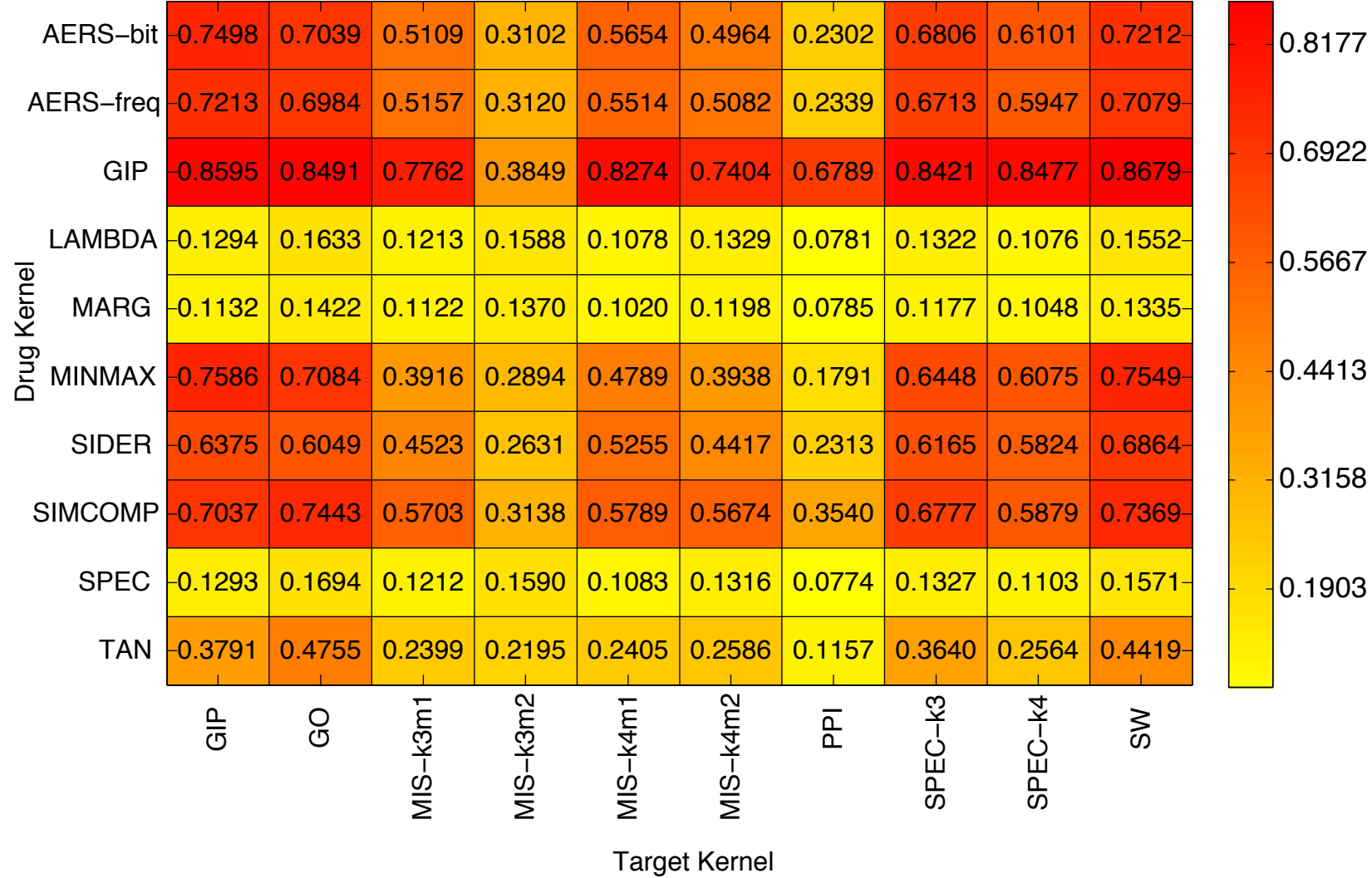

Enzyme

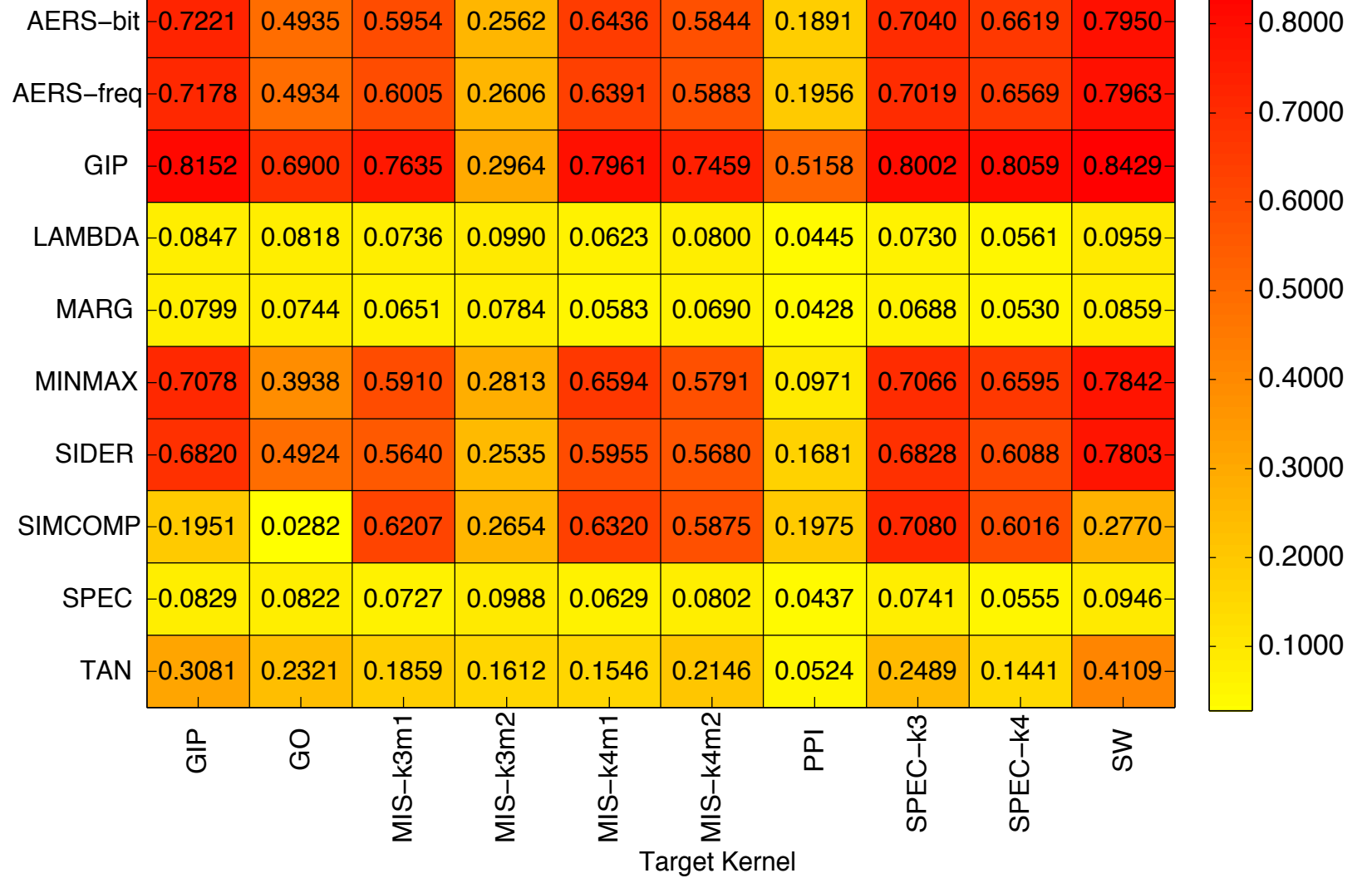

Supplement: Additional file 1 — Figure. Single kernel experiments on the Nuclear Receptor dataset with the KronRLS algorithm as base learner. The heatmap shows the AUPR performance of different kernel combinations; red means higher AUPR. (PDF 460 kb) [file 12859_2016_890_MOESM1_ESM.pdf]
