# Supplementary material for: A multiple kernel learning algorithm for drug-target interaction prediction
Source: BMC Bioinformatics. 2016 Jan 22;17:46. doi: 10.1186/s12859-016-0890-3 (PMC4722636; doi:10.1186/s12859-016-0890-3)
Supplement: Additional file 3 — Supplementary Tables. AUPR Results of competing methods under pair prediction setting considering subsampled test sets (S1); AUPR results of predicted scores against new interactions found on current release of KEGG, Matador, Drugbank and ChEMBL databases (S2); Average memory (MB) usage during training and testing of competing methods (S3); Average time (minutes) required to train and test the models with the competing methods (S4). (PDF 89.7 kb) [file 12859_2016_890_MOESM3_ESM.pdf]

Table S1: Results of competing methods under pair prediction in the subsampled test sets .

		Pairs	
Dataset	Combination	AUPR	
NR	[SPEC-k4]-[AERS-freq] †	0.7616	(±0.0205)
	[SPEC-k4]-[GIP] *	0.8389	(±0.0176)
	BLM-KA	0.5069	(±0.0198)
	BLM-MEAN	0.5070	(±0.0251)
	KBMF2MKL	0.6344	(±0.0163)
	KRONRLS-KA	0.8124	(±0.0281)
	KRONRLS-MEAN	0.8088	(±0.0275)
	KRONRLS-MKL	**0.8197**	(±0.0290)
	LAPRLS-KA	0.6993	(±0.0421)
	LAPRLS-MEAN	0.6891	(±0.0504)
	NETLAPRLS-KA	0.7075	(±0.0402)
	NETLAPRLS-MEAN	0.7033	(±0.0453)
	PKM-KA	0.7012	(±0.0416)
	PKM-MAX	0.5551	(±0.0263)
	PKM-MEAN	0.6982	(±0.0468)
	SITAR	0.7990	(±0.0442)
	WANG-MKL	0.7390	(±0.0317)
GPCR	[SPEC-k4]-[MINMAX] †	0.8703	(±0.0084)
	[SW]-[GIP] *	0.9249	(±0.0028)
	BLM-KA	0.6416	(±0.0195)
	BLM-MEAN	0.6234	(±0.0125)
	KBMF2MKL	0.8637	(±0.0098)
	KRONRLS-KA	0.9417	(±0.0039)
	KRONRLS-MEAN	0.9398	(±0.0046)
	KRONRLS-MKL	0.9440	(±0.0040)
	LAPRLS-KA	0.8204	(±0.0086)
	LAPRLS-MEAN	0.8206	(±0.0083)
	NETLAPRLS-KA	0.8593	(±0.0069)
	NETLAPRLS-MEAN	0.8604	(±0.0069)
	PKM-KA	0.8679	(±0.0083)
	PKM-MAX	0.7602	(±0.0167)
	PKM-MEAN	0.8631	(±0.0073)
	SITAR	**0.9470**	(±0.0065)
	WANG-MKL	0.9122	(±0.0067)
IC	[PPI]-[GIP] †	0.9372	(±0.0049)
	[SW]-[GIP] *	0.9815	(±0.0006)
	BLM-KA	0.7154	(±0.0122)
	BLM-MEAN	0.7049	(±0.0096)
	KBMF2MKL	0.9558	(±0.0020)
	KRONRLS-KA	0.9790	(±0.0011)
	KRONRLS-MEAN	0.9799	(±0.0008)
	KRONRLS-MKL	**0.9810**	(±0.0008)
	LAPRLS-KA	0.8721	(±0.0090)
	LAPRLS-MEAN	0.8756	(±0.0086)
	NETLAPRLS-KA	0.9218	(±0.0035)
	NETLAPRLS-MEAN	0.9252	(±0.0032)
	PKM-KA	0.9459	(±0.0057)
	PKM-MAX	0.8134	(±0.0080)
	PKM-MEAN	0.9501	(±0.0051)
	SITAR	0.9779	(±0.0021)
	WANG-MKL	0.9630	(±0.0027)
E	[GO]-[GIP] †	0.9632	(±0.0007)
	[SW]-[GIP] *	0.9777	(±0.0012)
	BLM-KA	0.7481	(±0.0066)
	BLM-MEAN	0.7431	(±0.0077)
	KBMF2MKL	0.9511	(±0.0019)
	KRONRLS-KA	0.9767	(±0.0007)
	KRONRLS-MEAN	0.9777	(±0.0005)
	KRONRLS-MKL	0.9804	(±0.0007)
	LAPRLS-KA	0.9106	(±0.0032)
	LAPRLS-MEAN	0.9068	(±0.0034)
	NETLAPRLS-KA	0.9187	(±0.0035)
	NETLAPRLS-MEAN	0.9158	(±0.0037)
	PKM-KA	0.9450	(±0.0040)
	PKM-MAX	0.8648	(±0.0027)
	PKM-MEAN	0.9412	(±0.0040)
	SITAR	**0.9885**	(±0.0012)
	WANG-MKL	0.9749	(±0.0014)

†best on training *best on testing

Table S2: AUPR results of predicted scores against new interactions found on current release of KEGG, Matador, Drugbank and ChEMBL databases.

Dataset	Combination	KEGG	MATADOR	DRUGBANK	ChEMBL
NR	SINGLE†	0.4122	0.0149	0.0442	0.2537
	SINGLE*	0.2533	0.0227	0.0389	0.2245
	BLM-KA	0.0069	0.0063	0.0108	0.1249
	BLM-MEAN	0.0048	0.0028	0.0079	0.1267
	KBMF2MKL	0.0394	0.0075	0.0128	0.2208
	KRONRLS-KA	0.0599	0.0095	0.0499	0.3246
	KRONRLS-MEAN	0.0410	0.0097	0.0469	0.3238
	KRONRLS-MKL	0.1235	0.0091	0.0472	0.3139
	LAPRLS-KA	0.0133	0.0104	0.0245	0.2949
	LAPRLS-MEAN	0.0133	0.0153	0.0297	0.3135
	NETLAPRLS-KA	0.0182	0.0130	0.0290	0.3071
	NETLAPRLS-MEAN	0.0176	0.0126	0.0277	0.3041
	NRWRH-KA	0.7600	0.0105	0.0351	0.1739
	NRWRH-MEAN	0.7600	0.0109	0.0365	0.1762
	PKM-MEAN	0.0094	0.0056	0.0358	0.2875
	PKM-KA	0.0132	0.0113	0.0183	0.2748
	PKM-MAX	0.0064	0.0053	0.0120	0.1728
	WANG-MKL	0.3403	0.0056	0.0430	0.3429
	SITAR	0.5204	0.0068	0.0618	0.2513
GPCR	SINGLE†	0.0912	0.0179	0.1481	0.2121
	SINGLE*	0.0912	0.0179	0.1481	0.2121
	BLM-KA	0.0118	0.0055	0.0423	0.1551
	BLM-MEAN	0.0116	0.0066	0.0447	0.1499
	KBMF2MKL	0.0586	0.0084	0.0831	0.1690
	KRONRLS-KA	0.0867	0.0230	0.1769	0.2489
	KRONRLS-MEAN	0.0746	0.0249	0.1880	0.2544
	KRONRLS-MKL	0.1085	0.0231	0.2191	0.2256
	LAPRLS-KA	0.0164	0.0129	0.0641	0.2508
	LAPRLS-MEAN	0.0182	0.0116	0.0656	0.2526
	NETLAPRLS-KA	0.0260	0.0199	0.0975	0.2606
	NETLAPRLS-MEAN	0.0269	0.0205	0.1004	0.2606
	NRWRH-KA	0.1555	0.0110	0.1871	0.2000
	NRWRH-MEAN	0.1527	0.0109	0.1861	0.1947
	PKM-MEAN	0.0214	0.0377	0.0639	0.2446
	PKM-KA	0.0252	0.0085	0.0792	0.2361
	PKM-MAX	0.0159	0.0134	0.0360	0.1682
	WANG-MKL	0.0691	0.0249	0.1651	0.2469
	SITAR	0.1536	0.0097	0.2289	0.1997
IC	SINGLE†	0.0905	0.0234	0.0557	0.0409
	SINGLE*	0.0905	0.0234	0.0557	0.0409
	BLM-KA	0.0125	0.0131	0.0184	0.0219
	BLM-MEAN	0.0126	0.0127	0.0174	0.0171
	KBMF2MKL	0.0419	0.0255	0.0457	0.0421
	KRONRLS-KA	0.0714	0.0348	0.0472	0.0399
	KRONRLS-MEAN	0.0618	0.0275	0.0369	0.0363
	KRONRLS-MKL	0.0723	0.0261	0.0462	0.0403
	LAPRLS-KA	0.0105	0.0258	0.0151	0.0315
	LAPRLS-MEAN	0.0106	0.0256	0.0152	0.0316
	NETLAPRLS-KA	0.0221	0.0261	0.0217	0.0301
	NETLAPRLS-MEAN	0.0231	0.0257	0.0223	0.0300
	NRWRH-KA	0.1585	0.0255	0.0987	0.0272
	NRWRH-MEAN	0.1646	0.0254	0.1063	0.0271
	PKM-MEAN	0.0285	0.0238	0.0275	0.0455
	PKM-KA	0.0228	0.0218	0.0239	0.0631
	PKM-MAX	0.0080	0.0095	0.0134	0.0140
	WANG-MKL	0.0941	0.0438	0.0441	0.0595
	SITAR	0.0983	0.0264	0.0599	0.0319
E	SINGLE†	0.0058	0.0621	0.0513	0.0422
	SINGLE*	0.0058	0.0621	0.0513	0.0422
	BLM-KA	0.0047	0.0451	0.0116	0.0272
	BLM-MEAN	0.0049	0.0456	0.0118	0.0273
	KBMF2MKL	0.0034	0.0570	0.0311	0.0556
	KRONRLS-KA	0.0084	0.0666	0.0325	0.0385
	KRONRLS-MEAN	0.0063	0.0652	0.0376	0.0432
	KRONRLS-MKL	0.0106	0.0638	0.0445	0.0335
	LAPRLS-KA	0.0008	0.0041	0.0183	0.0599
	LAPRLS-MEAN	0.0007	0.0038	0.0179	0.0581
	NETLAPRLS-KA	0.0070	0.0354	0.0247	0.0641
	NETLAPRLS-MEAN	0.0070	0.0351	0.0241	0.0620
	NRWRH-KA	0.0125	0.0121	0.0268	0.0402
	NRWRH-MEAN	0.0098	0.0123	0.0247	0.0419
	PKM-MEAN	0.0001	0.0013	0.0024	0.0155
	PKM-KA	0.0001	0.0012	0.0028	0.0156
	PKM-MAX	0.0001	0.0009	0.0028	0.0180
	WANG-MKL	0.0001	0.0011	0.0025	0.0137
	SITAR	0.0006	0.0045	0.0322	0.0403

†best on training *best on testing

Table S3: Average memory (MB) usage during training and testing of competing methods.

Dataset	Combination	Pairs	Targets	Drugs
NR	BLM-KA	383.00	359.80	356.80
	BLM-MEAN	380.00	363.20	362.00
	KBMF2MKL	358.40	362.00	372.20
	KRONRLS-KA	367.00	355.60	356.60
	KRONRLS-MEAN	356.40	360.00	363.80
	KRONRLS-MKL	388.20	383.20	385.60
	LAPRLS-KA	367.60	367.00	360.80
	LAPRLS-MEAN	358.60	364.00	359.60
	NETLAPRLS-KA	384.67	365.80	359.80
	NETLAPRLS-MEAN	360.20	359.80	360.60
	NRWRH-KA	-	358.60	358.60
	NRWRH-MEAN	-	357.00	361.60
	PKM-KA	359.40	358.00	355.60
	PKM-MAX	358.20	355.00	360.80
	PKM-MEAN	358.80	358.60	358.60
	SITAR	370.40	373.20	369.20
	WANG-MKL	381.20	384.80	383.40
GPCR	BLM-KA	584.80	488.00	542.40
	BLM-MEAN	581.20	514.20	462.40
	KBMF2MKL	372.80	497.00	507.20
	KRONRLS-KA	370.40	563.20	570.80
	KRONRLS-MEAN	365.60	484.40	417.60
	KRONRLS-MKL	397.60	512.40	577.00
	LAPRLS-KA	415.40	403.20	512.60
	LAPRLS-MEAN	499.60	440.00	454.40
	NETLAPRLS-KA	370.60	382.40	381.20
	NETLAPRLS-MEAN	368.60	386.20	376.20
	NRWRH-KA	-	438.80	473.20
	NRWRH-MEAN	-	465.60	462.40
	PKM-KA	541.80	496.80	545.20
	PKM-MAX	512.60	635.80	600.40
	PKM-MEAN	525.60	617.80	522.00
	SITAR	450.40	428.80	490.60
	WANG-MKL	550.60	576.60	525.40
IC	BLM-KA	946.00	575.80	552.00
	BLM-MEAN	879.40	502.60	612.60
	KBMF2MKL	517.80	521.40	522.40
	KRONRLS-KA	476.20	469.80	469.00
	KRONRLS-MEAN	457.60	459.00	580.00
	KRONRLS-MKL	565.40	547.40	654.00
	LAPRLS-KA	497.00	417.00	544.80
	LAPRLS-MEAN	484.20	491.20	467.20
	NETLAPRLS-KA	384.40	382.40	381.20
	NETLAPRLS-MEAN	380.00	386.20	376.20
	NRWRH-KA	-	376.00	487.80
	NRWRH-MEAN	-	375.80	504.60
	PKM-KA	813.40	677.60	723.80
	PKM-MAX	788.80	680.20	855.40
	PKM-MEAN	805.40	695.60	861.00
	SITAR	540.40	384.80	567.80
	WANG-MKL	809.20	863.20	855.60
E	BLM-KA	3411.00	921.40	1021.20
	BLM-MEAN	3604.20	915.40	996.40
	KBMF2MKL	861.80	716.80	834.00
	KRONRLS-KA	734.80	675.60	784.80
	KRONRLS-MEAN	661.40	818.20	698.20
	KRONRLS-MKL	844.40	816.80	717.40
	LAPRLS-KA	641.20	705.60	709.40
	LAPRLS-MEAN	668.20	701.20	668.60
	NETLAPRLS-KA	562.60	567.80	576.80
	NETLAPRLS-MEAN	542.60	549.40	575.60
	NRWRH-KA	-	645.60	642.80
	NRWRH-MEAN	-	691.60	728.20
	PKM-KA	4899.20	4985.20	5220.20
	PKM-MAX	5048.20	4997.00	5195.40
	PKM-MEAN	4976.80	4891.60	5194.20
	SITAR	789.80	598.00	741.40
	WANG-MKL	4990.40	5090.60	5121.60

Table S4: Average computing time in minutes for training and testing the model in all four datasets (ranking of each method is given in parenthesis).

METHOD	PAIRS	TARGETS	DRUGS
BLM-KA	340.32 (15)	54.25 (13)	24.68 (10)
BLM-MEAN	299.60 (14)	39.95 (12)	16.24 (5)
KBMF-MKL	105.17 (11)	110.34 (15)	114.30 (15)
KRONRLS-KA	21.17 (7)	21.53 (6)	21.39 (8)
KRONRLS-MEAN	20.39 (6)	21.51 (5)	21.34 (7)
KRONRLS-MKL	74.00 (10)	92.82 (14)	93.25 (14)
LAPRLS-KA	27.11 (8)	33.82 (9)	33.80 (13)
LAPRLS-MEAN	30.28 (9)	33.40 (8)	33.37 (12)
NETLAPRLS-KA	135.20 (13)	134.64 (17)	134.98 (17)
NETLAPRLS-MEAN	132.16 (12)	132.65 (16)	132.75 (16)
NRWRH-KA	-	39.90 (11)	30.09 (11)
NRWRH-MEAN	-	38.36 (10)	23.03 (9)
PKM-KA	0.63 (3)	0.95 (4)	0.64 (3)
PKM-MAX	0.55 (2)	0.93 (3)	0.61 (2)
PKM-MEAN	0.54 (1)	0.84 (2)	0.54 (1)
SITAR	15.54 (5)	22.59 (7)	16.30 (6)
WANG-MKL	0.82 (4)	0.80 (1)	0.68 (4)
